# Sensory system plasticity in a visually specialized, nocturnal spider

**DOI:** 10.1038/srep46627

**Published:** 2017-04-21

**Authors:** Jay A. Stafstrom, Peter Michalik, Eileen A. Hebets

**Affiliations:** 1 University of Nebraska - Lincoln, School of Biological Sciences, NE, USA; 2 University of Greifswald, Zoologisches Institut und Museum, Germany

## Abstract

The interplay between an animal’s environmental niche and its behavior can influence the evolutionary form and function of its sensory systems. While intraspecific variation in sensory systems has been documented across distant taxa, fewer studies have investigated how changes in behavior might relate to plasticity in sensory systems across developmental time. To investigate the relationships among behavior, peripheral sensory structures, and central processing regions in the brain, we take advantage of a dramatic within-species shift of behavior in a nocturnal, net-casting spider (*Deinopis spinosa*), where males cease visually-mediated foraging upon maturation. We compared eye diameters and brain region volumes across sex and life stage, the latter through micro-computed X-ray tomography. We show that mature males possess altered peripheral visual morphology when compared to their juvenile counterparts, as well as juvenile and mature females. Matching peripheral sensory structure modifications, we uncovered differences in relative investment in both lower-order and higher-order processing regions in the brain responsible for visual processing. Our study provides evidence for sensory system plasticity when individuals dramatically change behavior across life stages, uncovering new avenues of inquiry focusing on altered reliance of specific sensory information when entering a new behavioral niche.

Sensory systems can differ dramatically in form and function across animal taxa. Though highly variable, sensory systems tend to match the sensory needs of a given animal. Sensory information most important for an animal’s survival and reproductive success, for example, tends to be associated with the most highly elaborate sensory or processing structures^1^,^2^. This seems to occur, in part, due to the high metabolic costs of developing and maintaining both peripheral (*e.g*. eyes) and central (*e.g*. visual processing centers) sensory structures^1^,^2^.

Comparative evidence from a diverse array of taxa highlights the role of environmental and behavioral variation on sensory system evolution. Large differences in sensory system investment, both in peripheral sensory structures and central nervous processing, can be observed among closely related animals that live in different environmental conditions^3^,^4^,^5^,^6^,^7^,^8^. A classic example is the blind Mexican cavefish, where populations that live permanently in caves, with little-to-no light, possess eyes and visual processing centers of reduced size, when compared to surface-dwelling species^3^,^4^,^5^. Similar reductions are seen in other cave-dwelling animals (Isopods^9^,^10^, Crayfish^11^,^12^).

In addition to the importance of the environment on sensory system evolution, an animal’s behavior can also influence sensory system form and function^7^,^8^,^13^,^14^,^15^. For example, paper wasp species that use unique facial markings for individual recognition are more likely to have larger eyes with larger facets, presumably allowing for better recognition, when compared to species without visual markings^15^. Even within species, we see differences in behavior reflected in sensory system variation^16^,^17^,^18^,^19^,^20^,^21^,^22^.

The extent to which sensory systems vary, both across and within species, suggests high levels of plasticity, or the ability to alter investment; but the degree to which an individual’s sensory system is plastic is less explored (but see refs 18 and 23). To what extent can an individual’s sensory system change over time in response to changing conditions/contexts? Investigations into the potential for dynamic sensory system changes within individuals across development, especially in species where individuals dramatically alter their environmental/behavioral needs throughout their lives, could greatly expand our understanding of sensory system plasticity, constraints, and trade-offs. To this end, we leverage the unusual natural history of a net-casting spider (Order Araneae, Family Deinopidae) to assess the relationship between peripheral sensory structures (eyes) and underlying neural tissue across developmental time (penultimate vs. adult) and sex (female vs. male). Specifically, we use natural variation in foraging behavior and eye size observed across sex and life stage in the net-casting spider, *Deinopis spinosa*^24^, to investigate the relationship between eye diameter, relative investment in sensory processing, and potential trade-offs between lower-order and higher-order processing centers in the brain.

*Deinopis* spiders are known for their unusual nocturnal foraging strategy termed ‘net-casting’- actively entangling prey using a specialized capture snare^25^; and all *Deinopis* exhibit net-casting prior to sexual maturation. Following maturation, however, females continue to actively forage while males are no longer capable of net-casting – they lose the specialized structures on their legs (calamistrum) required to create the capture snare silk^26^. In addition to this unusual hunting strategy, spiders within the genus *Deinopis* also possess extraordinarily enlarged Posterior Median Eyes (PMEs; Fig. 1). These PMEs are the largest eyes of any spider^26^; they possess huge photoreceptors (20 μm wide, 110 μm long^27^); and they have a very short focal distance^26^. Together, these traits are presumed to increase the spider’s sensitivity to light and thus aid in nocturnal foraging^25^,^26^. Indeed, recent field and laboratory studies that reversibly occluded the PMEs of foraging *Deinopis* supported this presumption as the PMEs were shown to aid in nocturnal foraging of cursorial (ground-dwelling) prey items^28^. Coincident with the loss of net-casting in mature male *Deinopis*, PMEs appear reduced in size (Fig. 1). In *D. subrufa*, the size of the PMEs of mature males was shown to decrease up to 25% in diameter in relation to their penultimate morphology^26^.

Most spiders have eight eyes divided into one anterior and one posterior row of four each^29^. Within each row are pairs of median and lateral eyes. The median eyes of the anterior row (Anterior Median Eyes, hereafter AMEs) are known as the principal eyes. In these eyes, the distal segment of the photoreceptor, which is closest to the lens, contains photoreceptive pigment, while the cell body is more proximal^30^. The principal eyes are always forward facing and are image-forming in most web-spinning and ground-dwelling spiders, though typically of coarse resolution^27^. However, in jumping spider (Family: Salticidae), these eyes are enlarged and are well-adapted for high resolution vision in bright light environments^31^. The remaining six eyes are known as the secondary eyes, and are physiologically and functionally different from the principal eyes. In these eyes, the distal-most photoreceptor segment contains the cell body, while the photoreceptive pigment lies proximally, closer to the optic nerve^30^. Secondary eyes can be oriented to capture peripheral fields of view^27^ and typically possess a mirror-like tapetum behind the retina that reflects light back through photoreceptors^27^. The secondary eyes are regarded as motion-detectors^27^, and can also aid in navigation guided by the sun or other celestial cues^30^. Principal and secondary eyes not only function differently, but also have distinct connectivity in the brain (Fig. 2). While information from both eye types pass through respective lower-order visual processing centers (Optic Neuropils, hereafter ONPs)^32^,^33^,^34^, visual information from the secondary eyes is directed towards the mushroom bodies (hereafter MBs)^32^,^34^, while the arcuate body (hereafter AB) receives direct information from the principle eyes^32^,^33^ and likely indirect input from the secondary eyes^34^. While both are regarded as higher-order integration centers, spider MBs are functionally distinct from insect MBs^32^,^35^, as olfactory processing has not been shown to take place in spider MBs^32^,^35^. The AB also has similarities to an insect brain region (the central body), as both are integration centers likely responsible for motor control^32^, yet their homology remains debated^32^,^36^,^37^.

Ultimately, foraging behavior (presence of net-casting) as well as peripheral sensory system morphology (size of PMEs) are known to vary dramatically across penultimate to adult life stages in male *Deinopis* spiders. This natural variation in specialized sensory structure size and associated behavior across sex and life stage provides a unique opportunity to explore the potentially dynamic, within-individual relationship between peripheral sensory structures and central sensory system processing. Our study addressed three major objectives. First, we compared variation of peripheral visual sensory structure size (PME and AME diameter) across sex (male vs. female) and life stage (penultimate vs. mature). If sensory system structure is tightly linked to the specialized net-casting behavior in *D. spinosa*, then we expect mature males to possess the smallest absolute and relative PMEs. As no functional importance has yet been ascribed to the principal eyes of *Deinopis*, AME size is not expected to significantly differ across groups. Second, if variation in either PMEs or AMEs exists across sex and/or life stage, neural investment dedicated to processing respective visual information should match peripheral sensory structure size. Thus, we aimed to quantify and compare the volume of all lower-order visual processing centers (ONPs), as well as two higher-order integration centers receiving direct information from either the PMEs or AMEs (*e.g*. the MBs or the AB, respectively). As the PMEs are significantly larger than the AMEs, we expect variation in PME size to better predict size of lower-order ONPs. We also expect a positive relationship between PME size and MB relative investment, as well as between AME size and AB relative investment. Lastly, we aimed to more directly elucidate potential trade-offs between neural tissue associated with distinct processing (lower-order visual vs. higher-order integration) by comparing the relative size of the ONPs to the relative sizes of the MBs and the AB separately across all focal groups (male/female and penultimate/mature). As previous studies have found evidence of trade-offs between lower-order and higher-order processing^16^, we expected to uncover similar evidence of trade-offs between investment in ONPs and higher-order integration centers.

## Results

### Peripheral sensory structure size between groups

#### Posterior Median Eyes (PMEs) - Absolute and relative eye size

**Figure 3:**
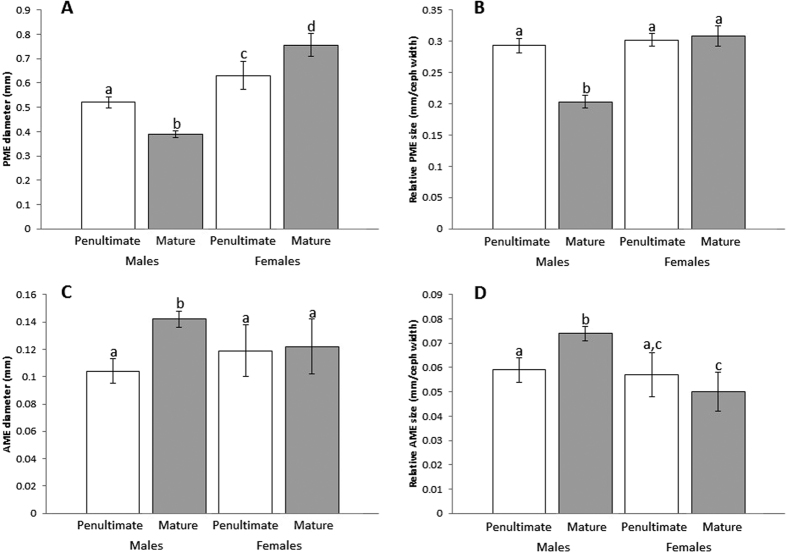
Eye size comparisons across groups. Significant differences are symbolized by differences in letters above respective bars. Absolute PME diameter (**A**) significantly differs across each group. While standardizing for body size using cephalothorax width measurements (**B**), mature males had relatively smaller PMEs than every other group, while no other group significantly differed from each other. Similarly, mature males had significantly larger absolute AME diameters (**C**) when compared to all other groups, while no other group was significantly different from each other. When standardizing for body size using cephalothorax measurements, mature males also had relatively larger AMEs than all other groups. Error bars signify one standard deviation above and below the average trait value.

Absolute PME size differed significantly when compared across all groups (Kruskal-Wallis: N = 39, χ^2^ = 34.602, P < 0.0001; Fig. 3A). Separate Mann Whitney U tests show absolute PME diameter significantly differs between all groups (See Supplementary Table S1). Specifically, females tended to have larger absolute PMEs than males, mature females had the largest PMEs, and mature males possessed the smallest PMEs of any focal group.

While controlling for body size (*i.e*. standardizing by cephalothorax width), relative PME size differed significantly when compared across all groups (Kruskal-Wallis: N = 39, χ^2^ = 24.715, P < 0.0001; Fig. 3B). Separate Mann Whitney U tests show that mature males had significantly smaller relative PME diameter when compared to every other group, while no other group significantly differed from each other in relative PME diameter (See Supplementary Table S1). Thus, mature males had the smallest relative PMEs, while all other groups possessed similarly sized relative PMEs.

#### Anterior Median Eyes (AMEs) - Absolute and relative eye size

Absolute AME size differed significantly when compared across all groups (Kruskal-Wallis: N = 39, χ^2^ = 19.921, P < 0.0001; Fig. 3C). Separate Mann Whitney U tests show that mature males possessed AMEs significantly larger in absolute diameter when compared to every other group, while no other group significantly differed from each other in absolute AME diameter (See Supplementary Table S1). Thus, mature males possessed the largest absolute AMEs, while all other groups had similarly sized AMEs.

While controlling for body size, relative AME size differed significantly when compared across all groups (Kruskal-Wallis: N = 39, χ^2^ = 24.47, P < 0.0001; Fig. 3D). Similar to absolute AME size, mature males possess the largest AMEs of relative size, when compared across all groups, while correcting for multiple comparisons (See Supplementary Table S1).

### Lower order sensory processing regions and higher order integration centers between groups

**Figure 4:**
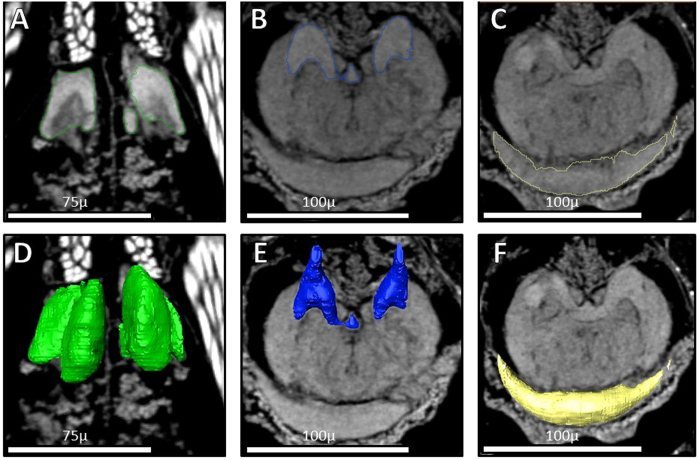
Micro-CT scans of focal brain regions. Dorsal views of segmented brain region outlines (**A**–**C**) and reconstructed regions (**D**–**F**). Focal regions depicted are the ONPs (**A**,**D**), the MBs (**B**,**E**), and the AB (**C**,**F**).

All spiders possessed central nervous systems of a generally similar layout (Fig. 4). Each focal brain region was bilaterally symmetric, and in similar placement within the central nervous system across all groups investigated. Absolute brain size differed significantly across all groups (Kruskal-Wallis: N = 40, χ^2^ = 9.258, P = 0.026), where mature spiders typically had larger brains than penultimate spiders (See Supplementary Table S2).

#### Absolute and relative investment in lower-order optic neuropils (ONPs)

**Figure 5:**
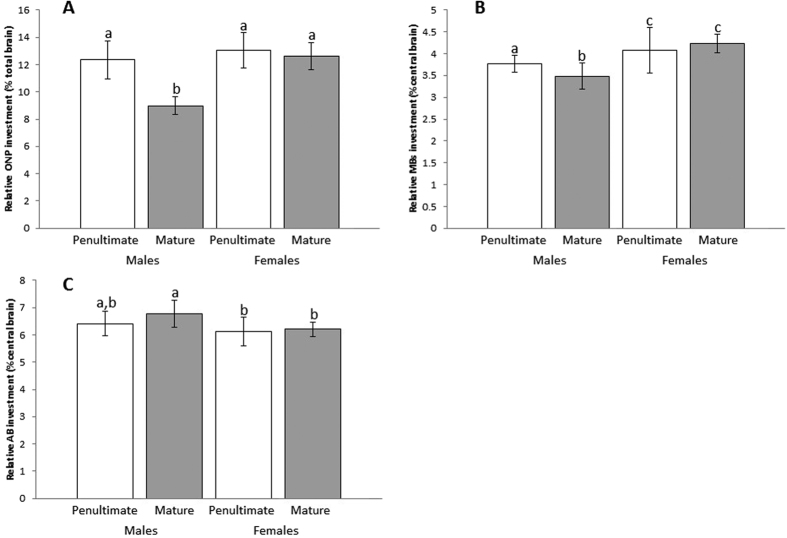
Neural structure comparisons across groups. Significant differences are symbolized by differences in letters above respective bars. Relative ONP investment (**A**), standardized using total protocerebrum volume, is significantly lower in mature males when compared to every other group, while all other groups had similar relative investment in ONPs. Relative investment in the MBs (**B**), standardized using “central brain” total volume, was significantly greater in females overall, and lowest in mature males. Relative investment in the AB (**C**), also standardized using “central brain” volume, was highest in mature males and was significantly greater in mature males than in both penultimate and mature females. Error bars signify one standard deviation above and below the average trait value.

Absolute size of lower-order ONPs differed across groups (Kruskal-Wallis: N = 40, χ^2^ = 8.437, P = 0.038; See Supplementary Table S2). Relative investment of lower-order ONPs also differed significantly across groups (Kruskal-Wallis: N = 40, χ^2^ = 21.830, P < 0.0001; Fig. 5A). Mature males had the smallest relative investment in ONPs, while penultimate males, penultimate females, and mature females had similar patterns of relative investment. Separate Mann Whitney U tests show that mature males had the smallest relative ONPs, while no other comparisons were significant (See Supplementary Table S2).

#### Relative investment in higher-order integration neuropils (MBs and AB)

Relative investment in the MBs differed significantly across groups (Kruskal-Wallis: N = 40, χ^2^ = 20.112, P < 0.0001; Fig. 5B). When corrected for multiple comparisons, separate Mann Whitney U tests show that mature males invested relatively less in MBs when compared to every other group (See Supplementary Table S2), and that penultimate males invested relatively less than both penultimate females (U = 22, P = 0.034) and mature females (U = 4, P = 0.001). Relative MB investment did not differ between penultimate and mature females (U = 39, P = 0.832).

Relative investment in the AB differed significantly across groups (Kruskal-Wallis: N = 40, χ^2^ = 9.809, P = 0.020; Fig. 5C). While separate comparisons showed that mature males had higher relative investment than both penultimate females (U = 15, P = 0.008) and mature females (U = 15, P = 0.008), no significant difference was found between penultimate and mature males when corrected for multiple comparisons (U = 19, P = 0.049). No other groups significantly differed (See Supplementary Table S2).

### Relationships between lower-order visual processing and higher-order integration centers

**Figure 6:**
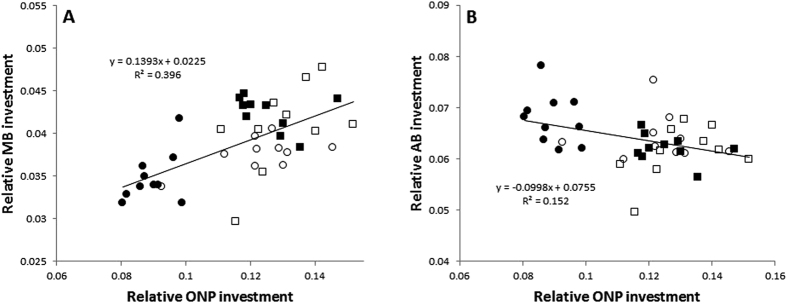
Relationships between relative investment in lower-order visual processing and higher-order integration centers across all focal groups (circles = male, square = female, white = penultimate, black = mature). A positive relationship exists between relative ONP investment and relative investment in the MBs (**A**). In contrast, a negative relationship exists between relative ONP investment and relative AB investment (**B**). Both relationships are heavily influenced by mature male investment.

Across all spiders, we found a positive relationship between the relative size of the ONPs and the MBs (Linear regression: *df* = 1, *F* = 24.911, R^2^ = 0.396, P = 0.001; Fig. 6A). In contrast, relative ONP investment was negatively correlated with investment in the AB (Linear regression: *df* = 1, *F* = 6.790, R^2^ = 0.152, P = 0.01; Fig. 6B).

## Discussion

In association with a previously established shift in ecology and behavior across life stages in net-casting spiders - in which mature males no longer engage in an unusual foraging strategy (*i.e*. net-casting) - we find associated changes in peripheral sensory system structure as well as relative lower and higher-order central nervous system processing investment. Mature male *D. spinosa* possess decreased posterior median eyes (PMEs) and enlarged anterior median eyes (AMEs) compared to penultimate males, penultimate females, and mature females. Central investment matches peripheral alterations; mature males invest relatively less in lower-order optic neuropils (ONPs) and the mushroom bodies (MBs) - a higher-order integration center that receives direct input from the PMEs, but not the AMEs^32^,^33^,^34^. When compared to penultimate and mature females, mature males also possess relatively larger arcuate bodies (AB) – a higher-order integration center receiving direct input from the AMEs, and likely indirect input from the PMEs^32^,^33^,^34^. Lastly, we describe a species-wide, positive relationship between relative investment in ONPs and the MBs, while also uncovering a negative relationship, and potential trade-off, in relative investment between the ONPs and the AB.

We first confirm a decrease in PME diameter among mature male *D. spinosa*, when compared to their penultimate stadium, reinforcing previous research showing similar reductions in an Australian net-casting spider, *D. subrufa*^26^. Similar to *D. subrufa*, the absolute size of mature male PMEs in *D. spinosa* decreases by ~25%. The PMEs in *D. spinosa* have recently been shown to be important in nocturnal foraging^28^. As mature males no longer engage in net-casting, they no longer rely on these enlarged eyes for this foraging function and coincident with this, we observe a decrease in PME diameter. Blest and Land^26^ found that decreased PME diameters relate to a decrease in retinal size, as mature male *D. subrufa* possess a retina of smaller area, comprised of smaller photoreceptors. While we would predict retinal area to match PME diameter in *D. spinosa*, and that photoreceptor size varies across groups, these aims were unfortunately beyond the scope of this study. These predictions remain to be studied in *D. spinosa*.

Consequent to a decrease in PME diameter, we found that mature males possess AMEs with increased diameter when compared to all other focal groups. The AMEs are image-forming in most web-spinning and ground-dwelling spiders, though typically of low quality resolution^27^, while jumping spiders possess extraordinary AMEs capable of forming highly resolved images^30^,^31^. However, nothing is currently known about the potential function of the enlarged AMEs in mature male *D. spinosa*. While the role of the principal eyes has remained unstudied in *Deinopis*, the discovery of enlargement of AMEs in mature males suggests a potentially important function of vision for these spiders; and specifically, the type of vision provided by the AMEs. Though mature males no longer rely on PME vision to forage, they may benefit from AME vision while searching for mates. Given that males actively wander in search of mates upon sexual maturation (pers. obs.), enlarged AMEs may aid mature males in locating and/or courting females through enhanced detail discrimination. Another possibility is that AMEs aid males in predator detection. The leg morphology and diurnal posture of mature male *D. spinosa* is less cryptic than that of mature females and/or juveniles (pers. obs.), potentially increasing selection on effective predator detection. These hypotheses remain to be tested.

In conjunction with differences in peripheral sensory morphology, specifically the reduction in PME diameter, we found a corresponding decrease of relative investment in lower-order visual processing in mature males. When compared to all other focal groups mature males had the lowest relative investment in ONPs, while all other spiders had similar degrees of investment. Neurons are among the most metabolically expensive bodily tissues; as neural tissue requires nearly a magnitude more energy per unit weight to function than most other tissue^38^,^39^,^40^. Additionally, apart from the energetic costs associated with neural function, metabolic costs are also high regarding neural tissue’s maintenance^4^, leading to an expectation of decrements in brain regions of decreased importance. This is exactly what we observe in *D. spinosa*, as a decrease in PME diameter is associated with a decreased relative investment of ONPs. Similar within-individual changes in neural investment have been observed in other animals. Seasonal changes in the brains of song birds, for example, are a well-documented case neural plasticity in which brain regions associated with call production become greatly reduced in the winter months when song production ceases^41^,^42^,^43^,^44^. For mature male *D. spinosa*, decreasing investment in PME size and processing could benefit males by saving energy to increase longevity or devote to locomotion, thus increasing the chances of finding a mate; and/or by freeing up resources to invest in other, newly important bodily tissue and/or brain regions.

In addition to our observed differences in ONP volume for mature males, relative volume of both of our focal higher-order integration brain regions (MBs and AB) also differed by sex, life stage, or both. As the MBs only receive direct input from the secondary eyes^34^, we expected PME size to predict relative investment in the MBs, and our analyses matched our predictions. Mature males had the smallest relative MBs, and both penultimate and mature females had greater relative investment in MBs than penultimate males, results that mirror PME size. As females possess larger PMEs than age-matched males, increased relative investment in the MBs might purely be dictated by processing from PMEs. While spider MBs are termed “higher-order integration regions”^32^, in contrast to insect mushroom bodies^35^, the functional relevance of spider MBs on behavior is not well-understood^32^. As such, it is difficult to further interpret our observed differences in relative MB size between male and female *D. spinosa*.

Similar to the MBs, we also documented differences in relative AB investment across *D. spinosa*. Relative investment in the AB was greatest in mature males and significantly greater in mature males than in penultimate and mature females spiders. While the AB likely receives information from the secondary eyes indirectly^34^, it is the only higher-order integration center to receive direct input from the AMEs^32^,^33^,^34^. Thus, similar to the MBs, increased relative investment in the AB could be partially explained by an increase in respective peripheral sensory structures (*i.e*. AMEs). As mentioned previously, we do not currently know what the function(s) might be of the larger AMEs of mature males. However, beyond AME visual information, the AB also receives inputs from midbrain neurons, potentially leading to a multi-modal integration role^32^. Mate-searching in spiders is thought to be predominantly chemically-based^45^,^46^,^47^,^48^. If the AB is, in part, responsible for processing and integrating chemical cues, increased importance of chemical processing in mate-searching males could explain AB investment differences in *D. spinosa*. To investigate such a hypothesis, it will be important for future studies to quantify the details of chemical processing pathways in net-casting spiders, as well as to explore male reliance on chemical cues for mate-searching. Additionally, it will be important to keep in mind that the AB is also presumed to be a motor control center^32^, and might function in this capacity as mature males are actively searching for mates. In any sense, future investigations focusing on the AB’s possible role in mate-searching have great potential for yielding interesting and valuable neuroethological results.

In addition to the previously discussed observed differences across life stage and sex with respect to specific processing centers (*i.e*. the ONPs, MBs, and AB), we also see relationships among these centers that suggest potential synergies, as well as investment trade-offs. Specifically, when comparing relative ONP size with relative MB and AB size, we unveiled contrasting relationships. Relative ONP volume was positively correlated with relative MB volume, while relative ONPs volume was negatively correlated with relative AB volume. Though MBs and the AB are higher-order integration centers, they differ in their connections with visual inputs (among others)^33^,^34^. In *Cupiennius* spiders, the MBs are a third-order optic neuropil that integrates visual information from the secondary eyes; they do not receive direct inputs from the AMEs^33^,^34^. If lower and higher-order processing of vision from the same eyes are positively related, then the positive relationship between relative investment in the ONPs and the MBs points to a high degree of processing of secondary eye vision in the ONPs. As the PMEs are the largest eyes of *D. spinosa*, the bulk of the ONPs are likely associated with their processing. Thus, a positive relationship between the ONPs and the MBs might be expected. In contrast, the AB in *Cupiennius* receives direct inputs from the AMEs^33^. Thus, if the ONPs are mostly dedicated to PME processing, this potential trade-off between relative investment in ONPs and the AB might infer a trade-off between distinct types of visual processing (principal vs. secondary).

Following Healy and Rowe^49^, the current study makes use of intra-specific comparisons, across similarly sized individuals, regarding relative investment in sensory processing. Such comparisons forego multiple difficulties common to comparative neuroanatomy when comparing brain volume across individuals. However, the current study can be regarded as a first step towards better understanding the neuroethology of spiders, and net-casting spiders, specifically. Volumetric analysis is a valuable method in informing researchers where to focus more detailed investigations. As such, our study created various new hypotheses concerning both behavior and neuroanatomy, illustrating the utility of such research. As well, our study displays μCT scanning as a newly-emerging methodology ideal to supplement classical neurohistological methods^50^. This relatively new method of scanning arthropod internal anatomy has great potential for yielding fast, accurate results across many understudied taxa, opening the door to much greater understanding of how neural systems have evolved.

In summary, we have documented plasticity in both peripheral sensory structures and associated processing centers in the net-casting spider *Deinopis spinosa*. This work provides an important foundation for more directed hypothesis-testing concerning functional benefits of distinct sensory structures (*e.g*. AMEs), as well as brain regions (MBs and the AB). Though many questions remain unanswered – *e.g*. what is the function of the enlarged AMEs of mature male *D. spinosa*? *–* we have provided a solid groundwork upon which to build. The neuroethology of spiders remains largely unexplored, especially as compared to the plethora of neuroethological studies conducted on their arthropod relatives – the insects^18^,^51^,^52^,^53^,^54^,^55^– making foundational studies such as this incredibly valuable. We are encouraged by recent strides in spider brain research^56^,^57^,^58^,^59^.

Numerous studies have shed light on how sensory systems can evolve across animals contrasting in habitat and behavior. Interspecific comparisons have been important in producing hypotheses regarding sensory system evolution, while investigations using intraspecific comparisons of sensory systems across life-stage can shed light on the important, yet understudied, function of sensory system plasticity. Plasticity in both peripheral sensory structures and their underlying neural tissues likely benefit animals exhibiting extraordinary behavioral changes across development, allowing for increased efficiency regarding tissue investment, but how constrained is this developmental plasticity? Future studies such as this, that leverage unusual natural histories and non-model organisms, will contribute to our working knowledge of the costs, benefits, and potential constraints of developmental sensory system plasticity.

## Methods

### Study species

In April 2014, *D. spinosa* specimens (N = 40) were collected from Micanopy, Florida USA. Spiders were transported to the University of Nebraska-Lincoln, USA and housed in cylindrical, plastic enclosures (10 × 10 × 14 cm) atop granite slabs covered by filter paper. Spiders were allowed water *ad libitum*, fed one cricket twice a week, and were housed under 14:10 light conditions under a reversed light cycle. Ten spiders from each of our focal groups (penultimate male = 10, mature male = 10, penultimate female = 10, mature female = 10) were used. The penultimate stage is the life stage which directly precedes maturity. Spiders were classified visually, through absence/presence of penultimate/mature sexual genitalia. Individuals in each of their respective life stages were sacrificed 14–20 days following their previous molt. Spiders were sacrificed by removing the legs and abdomen and immediately transferring the remaining cephalothorax into fixative solution (100%, Prefer, Anatech). Following fixation, spiders were transported to the University of Greifswald, Germany for morphological (external and internal) analyses.

### External Morphology

Specimens were digitally photographed using a Zeiss SteREO Discovery.V20 microscope. Each spider was photographed twice, once focused on the PMEs and once focused on the AMEs. All spiders were photographed except for one mature female that could not be located. Digital images were imported to Adobe Photoshop CS4, where eye diameter was calculated using the line tool and information tab, and translated from points to absolute size in millimeters. Three measurements of each eye were taken and averaged. To calculate a measure of eye size relative to body size, cephalothorax width was also quantified using identical methods. Both absolute and relative measurements of eye size were used in our analyses.

### Internal Morphology

Following fixation, samples were dehydrated using a graded ethanol series and incubated in a 1% iodine solution (iodine, resublimated [Carl Roth GmbH 1 Co. KG, Karlsruhe, Germany] in 99.8% ethanol) overnight. After several washing steps in 99.8% ethanol, specimens were critical point dried by using the automated dryer Leica EM CPD300 (Leica Microsystems GmbH, Wetzlar, Germany). The drying protocol included slow CO_2_ admittance with a delay of 120 seconds, 18 exchange cycles (CO_2_: 99.8% ethanol), followed by a slow heating process and slow gas discharge (for more details see ref. 50). Finally, samples were fixed on an insect pin with glue from a hot glue gun and scanned with an Xradia MicroXCT-200 X-ray imaging system at 40 KV and 8 W. Voxel size was between 2.3 μm and 2.5 μm for each scanned sample.

Volume rendering of image stacks was performed by using Amira 5.4.5 (FEI Visualization Science Group, Burlington, USA) using the “Volren” function. Color maps were set to “Grey,” and the histograms were adjusted to individual image stacks properties. Additionally, the function “Filtered Oblique Slices” was applied for visualization of internal structures. To quantify volume focal regions, regions were visually identified and then “Segmented”. Segmentation was completed by separately outlining edges of focal brain regions, and moving through image stacks in all three planes of view (xy, xz, yz). Thus, each focal region was outlined at three different viewing planes, likely increasing the accuracy of volumetric analysis when compared to one plane quantification typically utilized following classical microtomy techniques. Volumetric measurements were obtained after segmentation of each brain region using the “Quantification: 3D analysis: Volume” function.

Three distinct brain regions in the protocerebrum were quantified for each spider: ONPs, MBs, and AB^32^. See Fig. 4 for visual representation of each brain region. The ONPs are located anterior to the rest of the protocerebrum and are dedicated to solely processing lower-order visual information^32^. We sum the volumes of all ONPs to reflect total investment in low-level visual processing. However, while quantifying neural investment for each eye would add to this study, and the literature in general, our major objective was to compare “relative visual investment” generally across groups. Thus, our scans were conducted at such a resolution that tracing individual neurons from each retina was not possible. As we did not conduct back-fills during this study, we cannot confidently say which ONP belongs to which pair of eyes. While some of the lower-order ONPs correlate better with PME size than other ONPs, we would like to remain conservative with our claims, and compare relative investment in all lower-order ONPs. The MBs are sensory integration centers located directly posterior to the ONPs^32^. The MBs receive direct information from the secondary eyes and likely other sensory modalities^32^. Spider MBs seem to process primarily visual information^32^, in contrast with the olfaction-based insect MBs^33^. The arcuate body is a crescent shaped higher-order integration center located superior and posterior to the rest of the brain^32^. The arcuate body receives direct information from the principal eyes and secondary eyes.

To standardize for body size, absolute volumes of each brain region were translated into relative measurements. Following quantification of the entire protocerebrum, each focal brain region’s absolute volume was divided by the total volume of the protocerebrum. Thus, for each objective regarding “relative investment” in neural processing, these measures of relative size (focal region/total protocerebrum) are utilized.

As lower-order visual processing ONPs differed significantly across groups, using “% total brain neuropil” as a measure of relative investment for other neuropils would likely affect outcomes through differences in ONPs alone. Thus, we subtracted ONPs from the total brain neuropil to construct analyses of relative investment within the “central brain neuropil”. The following analyses reflect relative investment of higher-order integration centers within the “central brain”.

### Statistical analyses

We used non-parametric Kruskal-Wallis tests to compare absolute and relative measures of eye size (PMEs and AMEs) across all groups. Kruskal-Wallis tests were also used to compare both absolute and relative sizes of focal brain regions (ONPs, MBs, and the AB) across groups. Non-parametric Mann-Whitney U tests were further utilized to compare eye and brain region sizes between all possible group pairs (Penultimate male – Mature male; Penultimate male – Penultimate female; Penultimate male – Mature female; Mature male – Penultimate female; Mature male – Mature female; Penultimate female – Mature female). We corrected for multiple comparisons using the Holm-Bonferroni correction.

To investigate potential trade-offs between lower-order visual processing and higher-order integration centers, linear regressions were conducted regressing relative investment of ONPs onto relative size of both the MBs and the AB, separately. All statistical tests were conducted using SPSS v20 software.

## Additional Information

**How to cite this article**: Stafstrom, J. A. *et al*. Sensory system plasticity in a visually specialized, nocturnal spider. *Sci. Rep.* **7**, 46627; doi: 10.1038/srep46627 (2017).

**Publisher's note:** Springer Nature remains neutral with regard to jurisdictional claims in published maps and institutional affiliations.

## Supplementary Material

Supplementary Materials

## Figures and Tables

**Figure 1:**
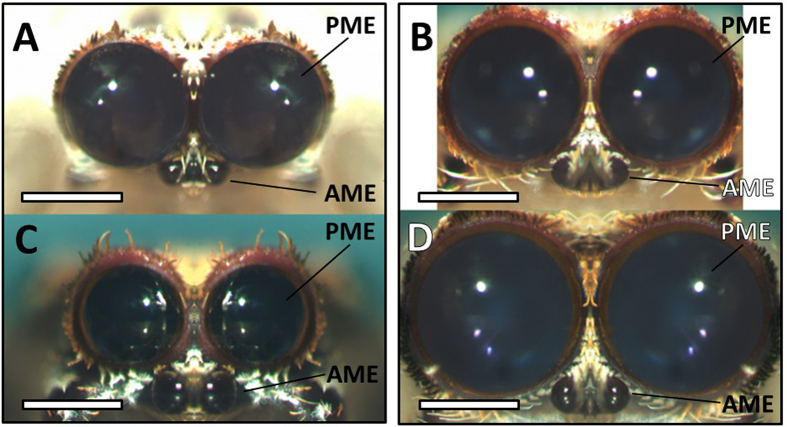
Forward facing eyes of *Deinopis spinosa* (PME = posterior median eye, AME = anterior median eye). Left and right columns represent photos from the same individual (A,C = male, B,D = female) across penultimate (**A**,**B**) and mature (**C**,**D**) life stages. All scale bars = 0.5 mm.

**Figure 2:**
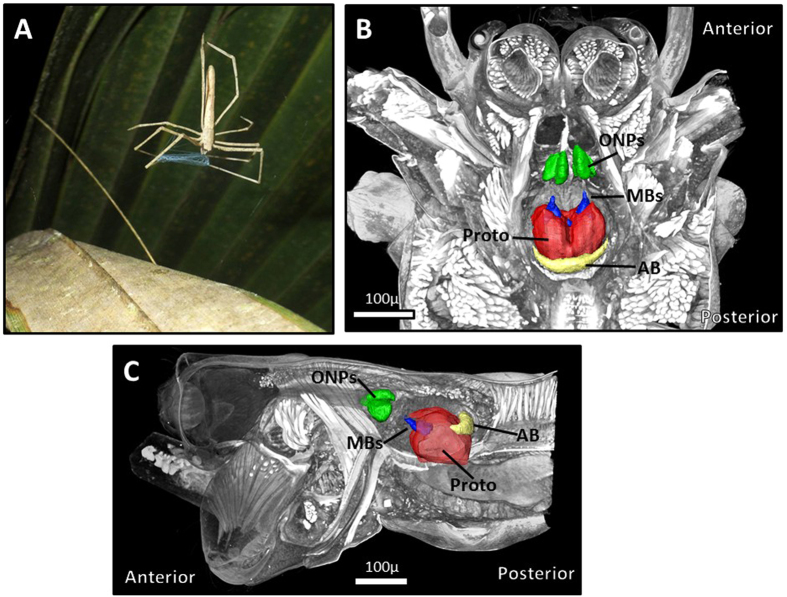
(**A**) *Deinopis spinosa* in foraging posture. While hanging in a support web (not detectable in this photograph), net-casting spiders will hold a specialized capture snare in their front legs, and actively entangle prey that walks beneath or flies above. (**B**,**C**) Internal anatomy within the cephalothorax of *D. spinosa*, (**B**) is a dorsal view, (**C**) is a lateral view. Protocerebrum (Proto) is red, ONPs are green, MBs are blue, AB is yellow. Anterior to the ONPs lies the optic nerve, which connects retinas from the eyes to the ONPs. The enlarged PMEs can be seen anterior to the optic nerve in (**C**).
