# Supplementary material for: Sensory system plasticity in a visually specialized, nocturnal spider
Source: Sci Rep. 2017 Apr 21;7:46627. doi: 10.1038/srep46627 (PMC5399460; doi:10.1038/srep46627)
Supplement: Supplementary Materials [file srep46627-s1.pdf]

SENSORY SYSTEM PLASTICITY IN A VISUALLY SPECIALIZED, NOCTURNAL SPIDER

Jay A. Stafstrom*[1], Peter Michalik[2], Eileen A. Hebets[1]

[1]University of Nebraska - Lincoln, School of Biological Sciences, NE, USA

[2]University of Greifswald, Zoologisches Institut und Museum, Germany

*Corresponding author:

Jay Stafstrom

Jstaf@huskers.unl.edu

Supplemental Table 1: Raw brain data of *Deinopis spinosa*.

All data quantified from segmented brains. Spider ID is the identifier for each sample. A focal spider's sex: 1=male, 2=female. A focal spider's stage represents its life stage: 1=penultimate, 2=mature. Raw data is recorded in μ^3 for the following: the lower-order optic neuropils (ONPs total), the arcuate body (AB), the mushroom bodies (MBs), and the protocerebrum (proto). Values are then summed (total brain) and used to standardize for size, by dividing the raw volume of each neuropil over the total brain (TB) volume. This is done for the optic neuropils (ONPs%), arcuate body (TB AB%), and the mushroom bodies (TB MBs %). Relative measures are given. To standardize for size within the central brain (CB), raw volumes were divided by (total brain - ONPs total). Relative measures are reported.

Spider ID	sex	stage	ONPs total	AB	MBs	Proto	total brain	ONPs %	TB AB %	TB MBs %	CB AB%	CB AB%
Ds-086-1	2	1	2631979.6	996080.2	597192.9	15906924	22764156.75	0.1156195	0.0437565	0.0262339	0.049477	0.0296636
Ds-085-1	1	2	1933719.2	1504713	713578.3	19499980	23651990.5	0.0817571	0.0636189	0.0301699	0.0692833	0.0328561
Ds-084-1	2	1	3176748.4	1315950	919462.7	20494300	25906461.1	0.1226238	0.0507962	0.0354916	0.0578956	0.040452
Ds-083-1	2	2	2482500.4	1145933	828413.9	16801160	21258007.3	0.1167795	0.0539059	0.0389695	0.0610334	0.0441221
Ds-082-1	2	1	2432937.6	991178.1	600692.7	13327520	17352328.4	0.1402081	0.0571208	0.0346174	0.0664356	0.0402625
Ds-081-1	1	1	2311702.7	1094011	685632	16505010	20596355.7	0.1122384	0.0531167	0.033289	0.0598322	0.0374977
Ds-080-1	1	2	1890809.2	1474089	688400.8	19437370	23490669	0.0804919	0.0627521	0.0293053	0.0682453	0.0318706
Ds-079-1	2	2	2638634.7	1265825	818678.5	17440730	22163868.2	0.1190512	0.0571121	0.0369375	0.0648302	0.0419293
Ds-078-1	1	1	2084242.2	1132371	596945.4	13319710	17133268.6	0.1216488	0.0660919	0.0348413	0.0752455	0.0396667
Ds-077-1	1	1	2068832.1	971990.8	539570.6	13436860	17017253.5	0.1215726	0.057118	0.0317073	0.065023	0.0360955
Ds-076-1	1	2	2563265.6	1833551	878397.3	23162660	28437873.9	0.0901356	0.0644757	0.0308883	0.0708629	0.0339482
Ds-075-1	1	1	2190396.4	1354092	723522.9	19378000	23646011.3	0.0926328	0.0572651	0.0305981	0.0631113	0.0337218
Ds-074-1	2	1	2466212	1071300	618374.2	15743050	19898936.2	0.1239369	0.053837	0.0310757	0.0614534	0.035472
Ds-073-1	1	2	2025438.5	1679667	725841.3	19090760	23521706.8	0.0861093	0.0714092	0.0308584	0.0781376	0.0337659
Ds-072-1	2	2	2764875.2	993621.9	675732.6	15950330	20384559.7	0.1356358	0.0487438	0.0331492	0.0563927	0.038351
Ds-071-1	2	2	3975142.2	1979642	1286178	26509020	33749982.2	0.1177821	0.0586561	0.038109	0.0664871	0.0431968
Ds-056-1	2	1	5144715.9	1718907	1177643	25822070	33863335.9	0.1519258	0.0507601	0.0347763	0.0598534	0.0410063
Ds-053-1	2	2	4652964.3	2113157	1474328	30472860	38713309.3	0.1201903	0.0545848	0.0380832	0.0620416	0.0432858
Ds-052-1	2	2	4306644	1889236	1302472	26938760	34437112	0.1250582	0.0548605	0.0378218	0.0627018	0.0432277
Ds-049-1	1	1	3925822.9	1676480	950216.6	23601790	30154309.5	0.1301911	0.0555967	0.0315118	0.0639183	0.0362284
Ds-047-1	1	2	3673203.8	2235325	1409642	30099870	37418040.8	0.0981667	0.0597392	0.0376728	0.066242	0.0417736
Ds-046-1	2	2	4132681.9	1482322	1052851	21400260	28068114.9	0.1472376	0.0528116	0.0375106	0.06193	0.0439871
Ds-045-1	2	2	4280580.6	1929643	1426936	28621070	36258229.6	0.1180582	0.0532194	0.0393548	0.0603435	0.0446229
Ds-041-1	1	1	2973260.2	1229141	766549.9	18079240	23048191.1	0.1290019	0.0533292	0.0332586	0.0612277	0.0381844
Ds-038-1	2	1	3057641.4	1376190	910390.7	18650050	23994272.1	0.1274321	0.0573549	0.037942	0.0657312	0.0434832
Ds-037-1	2	1	3624857.8	1440851	1058193	20243780	26367681.8	0.1374735	0.0546446	0.0401322	0.0633541	0.0465287
Ds-032-1	1	2	3352599.4	2051394	1128669	30043800	36576462.4	0.09166	0.0560851	0.0308578	0.0617446	0.0339716
Ds-031-1	1	1	3629035.5	1304500	815694.9	19155760	24904990.4	0.1457152	0.0523791	0.0327523	0.0613133	0.0383388
Ds-027-1	1	2	3295266.7	2271879	1203815	30981760	37752720.7	0.0872855	0.0601779	0.0318868	0.0659329	0.0349363
Ds-026-1	2	2	5468673.4	2327830	1456570	33009990	42263063.4	0.129396	0.0550795	0.0344644	0.0632659	0.0395867
Ds-024-1	1	2	4031572.1	2681763	1401343	33702720	41817398.1	0.096409	0.0641303	0.033511	0.0709727	0.0370865
Ds-019-1	2	1	5056765.8	1874984	1451250	27088110	35471109.8	0.1425601	0.0528595	0.0409136	0.061648	0.047716
Ds-018-1	2	2	5664282	2316606	1555419	33913380	43449687	0.1303642	0.053317	0.0357982	0.0613095	0.0411645
Ds-017-1	1	2	3781806.9	2133002	1096627	31199520	38210955.9	0.0989718	0.0558217	0.0286993	0.0619534	0.0318517
Ds-015-1	1	1	3168221	1483345	883916.3	19453300	24988782.3	0.1267857	0.0593604	0.0353725	0.0679792	0.0405084
Ds-014-1	1	1	2651270.8	1188295	726331.1	17143470	21709366.9	0.1221257	0.0547365	0.033457	0.0623512	0.0381114
Ds-010-1	2	1	3685872.4	1736559	1194994	26594070	33211495.4	0.1109818	0.0522879	0.0359813	0.0588153	0.0404731
Ds-008-1	2	1	4638352.2	2073928	1290942	27305750	35308972.2	0.1313647	0.0587366	0.0365613	0.0676194	0.0420905
Ds-006-1	1	1	3563150.5	1434572	886704.9	21186540	27070967.4	0.1316226	0.052993	0.0327548	0.0610253	0.0377196
Ds-003-1	1	2	2935474.6	1965629	1115563	27773740	33790406.6	0.086873	0.0581712	0.0330142	0.0637055	0.0361551

Supplemental table 2a: Kruskal-Wallis tests of absolute volume.

Absolute volumes of four focal neuropils (and the total brain) were compared using non-parametric Kruskal-Wallis tests. Proto=protocerebrum, total ONPs=all lower-order optic neuropils, mbs=mushroom bodies, ab=arcuate body. Sex: m=male, f=female. Stage: pen=penultimate, mat=mature. Avg=average, s.d.= standard deviation, sem=standard error mean, m.r.=mean rank, with χ^2 and P values following. Green shaded cells symbolize a significant difference across groups.

			absolute volume (*10^6 µm^3)				
Structure	sex	stage	avg	s.d.	sem	m.r.	χ^2 & P
total brain	m	pen.	23.027	4.129	1.306	12.20	
		mat.	32.467	7.040	2.226	26.60	9.258
	f	pen.	27.414	6.640	2.100	18.60	0.026
		mat.	32.075	8.635	2.731	24.60	
proto	m	pen.	18.126	3.205	1.013	12.20	
		mat.	26.500	5.638	1.783	28.00	11.077
	f	pen.	21.118	5.271	1.667	17.30	0.011
		mat.	25.106	6.764	2.139	24.50	
total ONPs	m	pen.	2.857	.695	.220	14.90	
		mat.	2.950	.805	.254	16.10	8.437
	f	pen.	3.592	1.036	.327	22.80	0.038
		mat.	4.037	1.116	.353	28.20	
mbs	m	pen.	.758	.131	.041	11.80	
		mat.	1.036	.272	.086	22.20	9.546
	f	pen.	.982	.305	.097	20.30	0.023
		mat.	1.188	.319	.101	27.70	
ab	m	pen.	1.287	.203	.066	11.70	
		mat.	1.983	.375	.119	29.80	14.062
	f	pen.	1.460	.381	.121	16.50	0.003
		mat.	1.744	.487	.154	24.00	

Supplemental table 2b: Kruskal-Wallis tests of relative volume, standardized by the entire brain.

Kruskal-Wallis tests were used to compare relative investment across four focal brain regions, standardized by the entire brain. All symbols are identical to those used in supplemental table 2a.

			relative volume (% total brain)				
			avg	s.d.	sem	m.r.	χ^2 & P
proto	m	pen.	78.749	1.415	.447	19.00	
		mat.	81.689	.773	.244	35.00	22.506
	f	pen.	76.944	2.804	.887	11.70	<0.0001
		mat.	78.280	.793	.251	16.30	
total ONPs	m	pen.	12.335	1.388	.439	23.90	
		mat.	8.979	.651	.206	5.80	21.830
	f	pen.	13.041	1.276	.403	28.00	<0.0001
		mat.	12.596	.985	.311	24.30	
mbs	m	pen.	3.295	.146	.046	15.60	
		mat.	3.169	.258	.082	10.90	17.220
	f	pen.	3.537	.428	.135	25.10	0.001
		mat.	3.701	.199	.063	30.40	
ab	m	pen.	5.620	.415	.131	20.00	
		mat.	6.164	.468	.148	32.70	16.049
	f	pen.	5.322	.432	.136	13.70	0.001
		mat.	5.423	.265	.084	15.60	

Supplemental table 2c: Kruskal-Wallis tests of relative volume, standardized by the central brain

Similar to table 2b, the depicted table utilizes brain regions standardized by the "central brain". Thus, instead of standardizing neuropils by the entire brain volume, we compare all brain regions except for the ONPs.

			relative volume (% central neuropil)				
			avg	s.d.	sem	m.r.	χ^2 & P
proto	m	pen.	89.830	.564	.178	25.30	
		mat.	89.747	.570	.180	22.40	3.654
	f	pen.	88.498	3.373	1.067	16.50	0.301
		mat.	89.562	.393	.124	17.80	
mbs	m	pen.	3.761	.191	.060	16.20	
		mat.	3.482	.297	.093	9.40	20.112
	f	pen.	4.071	.519	.164	25.50	<0.0001
		mat.	4.235	.207	.065	30.90	
ab	m	pen.	6.410	.457	.144	20.00	
		mat.	6.770	.497	.157	30.10	9.809
	f	pen.	6.122	.527	.166	15.60	0.020
		mat.	6.203	.270	.086	16.30	

Supplemental table 3: Mann-Whitney U tests

Mann-Whitney U tests comparing absolute, and two relative measures of brain region sizes across groups.

			absolute volume ($*10^6\ \mu m^3$)			relative volume (% central neuropil)			relative volume (% total neuropil)		
Structure	sex	life stage	m.r.	U	Z & P	m.r.	U	Z & P	m.r.	U	Z & P
total brain	m	juv.	7.00	15.00	2.646						
	m	mat.	14.00		0.008						
unspecified	m	juv.	6.60	11.00	2.948	13.60	42.00	0.605	6.00	5.00	3.402
protocerebrum	m	mat.	14.40		0.003	7.40		0.545	15.00		0.001
total ONPs	m	juv.	10.40	49.00	.076				15.20	3.00	3.553
	m	mat.	10.60		.940				5.80		<0.0001
mushroom	m	juv.	7.80	23.00	2.041	13.60	19.00	2.343	12.60	29.00	1.587
bodies	m	mat.	13.20		0.041	7.40		0.019	8.40		0.112
arcuate body	m	juv.	5.80	3.00	3.553	7.90	24.00	1.965	7.10	16.00	2.570
	m	mat.	15.20		<0.0001	13.10		0.049	13.90		0.010
total brain	m	juv.	8.50	30.00	1.512						
	f	juv.	12.50		0.131						
unspecified	m	juv.	8.90	34.00	1.209	12.20	33.00	1.285	12.90	26.00	1.814
protocerebrum	f	juv.	12.10		0.226	8.80		0.199	8.10		0.070
total ONPs	m	juv.	8.30	28.00	1.663				9.10	36.00	1.058
	f	juv.	12.70		0.096				11.90		0.290
mushroom	m	juv.	8.10	26.00	1.814	7.70	22.00	2.117	7.80	23.00	2.041
bodies	f	juv.	12.90		0.070	13.30		0.034	13.20		0.041
arcuate body	m	juv.	9.10	36.00	1.058	11.60	39.00	.832	12.30	32.00	1.361
	f	juv.	11.90		0.290	9.40		0.406	8.70		0.174
total brain	m	juv.	7.70	22.00	2.117						
	f	mat.	13.30		0.034						
unspecified	m	juv.	7.70	22.00	2.117	12.80	27.00	1.739	11.10	44.00	0.454
protocerebrum	f	mat.	13.30		0.034	8.20		0.082	9.90		0.650
total ONPs	m	juv.	7.20	17.00	2.495				10.60	49.00	0.076
	f	mat.	13.80		0.013				10.40		0.940
mushroom	m	juv.	6.90	14.00	2.721	5.90	4.00	3.477	6.20	7.00	3.250
bodies	f	mat.	14.10		0.007	15.10		0.001	14.80		0.001
arcuate body	m	juv.	7.80	23.00	2.041	11.50	40.00	.756	11.60	39.00	0.832
	f	mat.	13.20		0.041	9.50		0.450	9.40		0.406
total brain	m	mat.	12.70	28.00	1.663						
	f	juv.	8.30		0.096						
unspecified	m	mat.	13.30	22.00	2.117	11.80	37.00	0.983	15.50	0.00	3.780
protocerebrum	f	juv.	7.70		0.034	9.20		0.326	5.50		<0.0001
total ONPs	m	mat.	9.00	35.00	1.134				5.50	0.00	3.780
	f	juv.	12.00		0.257				15.50		<0.0001
mushroom	m	mat.	11.10	44.00	0.454	7.20	17.00	2.495	7.40	19.00	2.343
bodies	f	juv.	9.90		0.650	13.80		0.013	13.60		0.019
arcuate body	m	mat.	13.90	16.00	2.570	14.00	15.00	2.646	14.80	7.00	3.250
	f	juv.	7.10		0.010	7.00		0.008	6.20		0.001
total brain	m	mat.	10.90	46.00	0.302						
	f	mat.	10.10		0.762						
unspecified	m	mat.	11.30	42.00	0.605	11.90	36.00	1.058	15.50	0.00	3.780
protocerebrum	f	mat.	9.70		0.545	9.10		0.290	5.50		<0.0001
total ONPs	m	mat.	7.50	20.00	2.268				5.50	0.00	3.780
	f	mat.	13.50		0.023				15.50		<0.0001
mushroom	m	mat.	8.90	34.00	1.209	5.80	3.00	3.553	6.10	6.00	3.326
bodies	f	mat.	12.10		0.226	15.20		<0.0001	14.90		0.001
arcuate body	m	mat.	11.70	38.00	0.907	14.00	15.00	2.646	15.00	5.00	3.402
	f	mat.	9.30		0.364	7.00		0.008	6.00		0.001
total brain	f	juv.	8.80	33.30	1.285						
	f	mat.	12.20		0.199						
unspecified	f	juv.	8.50	30.00	1.512	9.50	40.00	0.756	9.10	36.00	1.058
protocerebrum	f	mat.	12.50		0.131	11.50		0.450	11.90		0.290
total ONPs	f	juv.	9.10	36.00	1.058				11.60	39.00	0.832
	f	mat.	11.90		0.290				9.40		0.406
mushroom	f	juv.	8.50	30.00	1.512	9.40	39.00	0.832	9.30	38.00	0.907
bodies	f	mat.	12.50		0.131	11.60		0.406	11.70		0.364
arcuate body	f	juv.	8.50	30.00	1.512	10.20	47.00	0.227	9.80	43.00	0.529
	f	mat.	12.50		0.131	10.80		0.821	11.20		0.597

Supplementary table 4a: Kruskal-Wallis tests of absolute and relative eye size

Absolute and relative (absolute eye size / cephalothorax width) sizes of both secondary (PMEs) and primary (AMEs) eyes. Sex: m=male, f=female. Stage: pen=penultimate, mat=mature. Avg=average, s.d.= standard deviation, sem=standard error mean, m.r.=mean rank, with χ^2 and P values following. Green shaded cells symbolize a significant difference across groups.

	sex	stage	avg (mm)	s.d. (mm)	sem (mm)	m.r.	χ^2 & P
**Absolute PME size**	m	pen.	0.520	0.024	0.007	15.90	
(mm)		mat.	0.389	0.015	0.004	5.50	34.602
	f	pen.	0.631	0.058	0.018	25.40	<0.0001
		mat.	0.756	0.047	0.016	34.67	
**Relative PME size**	m	pen.	0.293	0.012	0.003	20.40	
(abs/ceph width)		mat.	0.203	0.010	0.003	5.50	24.715
	f	pen.	0.302	0.010	0.003	25.70	<0.0001
		mat.	0.308	0.016	0.006	29.33	
**Absolute AME size**	m	pen.	0.104	0.009	0.003	9.70	
(mm)		mat.	0.142	0.006	0.002	32.20	19.921
	f	pen.	0.119	0.019	0.006	18.00	<0.0001
		mat.	0.122	0.020	0.007	20.11	
**Relative AME size**	m	pen.	0.059	0.005	0.002	19.50	
(abs/ceph width)		mat.	0.074	0.003	0.001	34.00	24.47
	f	pen.	0.057	0.009	0.003	16.40	<0.0001
		mat.	0.050	0.008	0.003	9.00	

Supplementary table 4b: Mann-Whitney U tests of absolute and relative eye size

Across each sex and life stage pair, comparisons of absolute and relative sizes of both PMEs and AMEs were conducted. Abbreviations remain consistent with previous supplemental tables.

		abs. PME size (mm)			relative PME size			abs. AME size (mm)			relative AME size		
sex	stage	m.r.	U	Z & P	m.r.	U	Z & P	m.r.	U	Z & P	m.r.	U	Z & P
m	pen.	15.50	0.00	3.780	15.50	0.00	3.780	5.50	0.00	3.780	5.50	0.00	3.780
m	mat.	5.50		<0.0001	5.50		<0.0001	15.50		<0.0001	15.50		<0.0001
m	pen.	5.90	4.00	3.477	8.30	28.00	1.663	8.20	27.00	1.739	11.60	39.00	0.832
f	pen.	15.10		0.001	12.70		0.096	12.80		0.082	9.40		0.406
m	pen.	5.50	0.00	3.674	7.60	21.00	1.960	7.00	15.00	2.451	13.40	11.00	2.776
f	mat.	15.00		<0.0001	12.67		0.050	13.33		0.014	6.22		0.006
m	mat.	5.50	0.00	3.780	5.50	0.00	3.780	14.30	12.00	2.873	15.00	5.00	3.402
f	pen.	15.50		<0.0001	15.50		<0.0001	6.70		0.003	6.00		0.001
m	mat.	5.50	0.00	3.674	5.50	0.00	3.674	13.40	11.00	2.776	14.50	0.00	3.674
f	mat.	15.50		<0.0001	15.50		<0.0001	6.22		0.006	5.00		<0.0001
f	pen.	5.80	3.00	3.429	8.50	30.00	1.225	9.50	40.00	0.408	12.00	25.00	1.633
f	mat.	14.67		0.001	11.67		0.221	10.56		0.683	7.78		0.102
